# Pressure injuries and biofilms: Microbiome, model systems and therapies

**DOI:** 10.1111/wrr.70005

**Published:** 2025-02-13

**Authors:** Fahad Kabir, Deborah Bow Yue Yung, Waleska Stephanie da Cruz Nizer, Kira Noelle Allison, Sandra Zigic, Emily Russell, Katrina G. DeZeeuw, Jonah E. Marek, Edana Cassol, Daniel Pletzer, Joerg Overhage

**Affiliations:** ^1^ Department of Health Sciences Carleton University Ottawa Ontario Canada; ^2^ Department of Microbiology and Immunology University of Otago Dunedin New Zealand; ^3^ Department of Complex Continuing Care Saint Vincent Hospital Ottawa Ontario Canada

**Keywords:** biofilms, chronic wounds, microbiome, pressure injury, treatment, wound healing

## Abstract

Chronic wounds have emerged as significant clinical problems owing to their increasing incidence and greater recognition of associated morbidity and socio‐economic burden. They are defined as wounds that do not progress normally through the stages of healing in a timely and/or orderly manner. Pressure injuries, in particular, represent a serious problem for patients who are elderly or have limited mobility, such as wheelchair users or those who spend most of the day in bed. These injuries often result from prolonged pressure exerted on the skin over the bone. Treatment of pressure injuries is complex and costly. Emerging evidence suggests that the pressure injury microbiome plays a vital role in chronic wound formation and delaying wound healing. Additionally, antibiotics often fail due to the formation of resistant biofilms and the emergence of antimicrobial‐resistant bacteria. In this review, we will summarise the current knowledge on: (a) biofilms and microbiomes in pressure injuries; (b) in vitro and in vivo model systems to study pressure injuries, and (c) current therapies and novel treatment approaches. Understanding the complex interactions between microbes and the host immune system in pressure injuries will provide valuable insights to improve patient outcomes.

**Abbreviations**
AgS: silver sulfadiazine
AMP: antimicrobial peptide
APCP: atmospheric pressure cold plasma
BHI: brain heart infusion
CFU: colony forming units
DFR: drip‐flow reactor
EPS: extracellular polymeric substances
HBOT: hyperbaric oxygen therapy
LCWB: lubbock chronic wound biofilm
PIs: pressure injuries
SBMA: sulfobetaine methacryate
TWOT: transdermal wound oxygen therapy

## 1 INTRODUCTION

Chronic wounds are a significant problem encountered by the ageing population, individuals with limited mobility, and those with certain pre‐existing health conditions like diabetes.^1^ Chronic wounds are defined as wounds that do not progress normally through the stages of healing in a timely and/or orderly manner.^2^ Patients often experience significant distress from these injuries (e.g., pain, bleeding, infection, decreased mobility), which can prolong hospitalisation and further their physical discomfort.^3^ If left untreated, wounds can lead to serious complications including cellulitis, gangrene or sepsis.^2^, ^4^ Chronic wounds also pose a significant issue to society as their treatment places an enormous financial burden on the healthcare system. For example, it has been estimated that such wounds cost the US healthcare system more than $28 billion each year.^5^

There are many different types of chronic wounds, with the four most common ones being diabetic foot ulcers, venous ulcers, arterial insufficiency ulcers and pressure injuries (PIs, also called pressure ulcers).^6^ These all have distinct physiologic characteristics such as their location on the body and aetiology.^7^ Treatments for these wounds have garnered increased attention due to increasing annual treatment costs,^8^ especially since many, including PIs, have become an increasingly common complication of ageing.^9^

**FIGURE 1:**
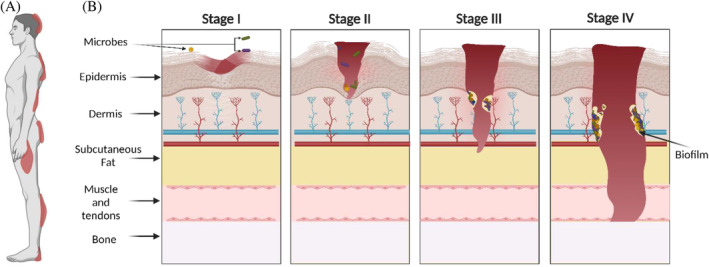
Location and stages of PI formation. (A) PIs typically form on ankles, back, elbow, heels, hips and shoulders. (B) PI stages (I–IV) increase in severity and extent of tissue damage as the stages increase.^10^, ^14^ Microbial species from the environment and normal microbiota occupy these areas and induce biofilms. *S. aureus*, *P. aeruginosa* and *S. epidermidis* are among the predominant species found within these biofilms and PIs.

As defined by the National Pressure Injury Advisory Panel, PIs develop ‘as a result of intense and/or prolonged pressure or pressure in combination with shear (horizontal stress whereby the bone moves across tissues and the skin is held in place)’.^10^, ^11^ Up to 30% of long‐term care residents^12^ and 40% of spinal cord injury patients experience some form of this injury.^13^ PIs can dramatically affect their quality of life and overall health outcomes. Common locations for PIs include the back/buttocks, elbow and heel of the foot^10^, ^14^ (Figure 1A). Prolonged pressure against a surface, such as a bed or chair can lead to PI development due to local tissue hypoxia and necrosis of skin tissue.^15^ This ischaemia along with shear/friction of the skin is the two main aetiologies of PIs.^16^ Additionally, contributing factors can increase an individual's risk of developing PIs, such as the integrity of blood vessels, health and nutritional status and pre‐existing health conditions like diabetes mellitus.^17^ Clinically, PIs are categorised into four stages according to the severity of tissue loss (Figure 1B), with each stage increasing in tissue damage depth.^17^ Stage I PI is an area of inflamed, intact skin that is not blanchable (i.e., skin colour does not change when pressed down).^15^ Stage II PI indicates damage to the superficial layers of the skin (epidermis, and sometimes dermis) often with tissue loss.^18^ Stage III PIs include the loss of full thickness of the skin and exposure of the underlying subcutaneous tissue^17^ including subcutaneous fat, but no tendon, muscle or bone. Stage IV PI involves severe tissue loss, complete necrosis of the skin, and exposure of muscle, tendon and/or bones.^18^ PI staging informs healthcare professionals about the severity of tissue damage and guides available treatment options.^15^ The cost of treating the wound increases with the stage of the PI as the wound requires more time to heal and complications are more probable (e.g., Stage III: $854 USD/day, Stage IV: $1785 USD/day).^19^

## 2 BIOFILMS AND PRESSURE INJURIES

**FIGURE 2:**
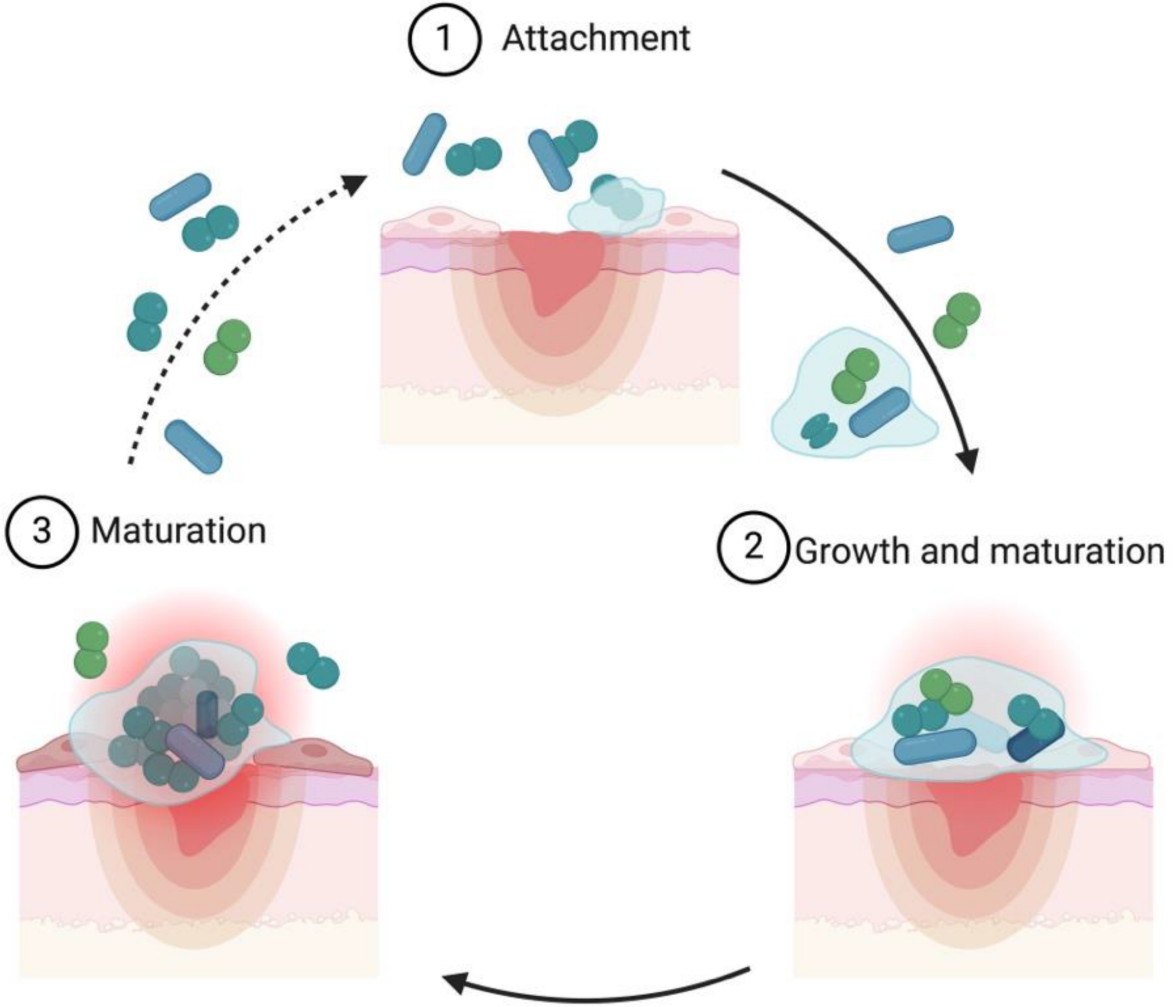
Proposed mechanisms of biofilm development in PI (adapted from Ref. [31]). Biofilm formation is a cyclic process that includes three main events, (1) attachment of bacterial cells; (2) biofilm growth and maturation and (3) detachment of single bacteria or aggregates from the biofilm.

The skin microbiota performs a vital role in protecting against the colonisation of pathogenic bacteria; furthermore, these commensals have been well documented to prevent dehydration, contribute to tissue repair, and enhance the host's immune response.^20^, ^21^ This first line of defence, created in tandem with skin commensals, prevents the colonisation of pathogenic bacteria through different avenues that entail the production of antagonistic chemicals (e.g., *Cutibacterium acnes* produces propionic acid), competition for nutrients, and altering the virulence of these environmental microbes.^21^ For instance, *Malassezia* spp. was observed to induce keratinocytes to produce pro‐inflammatory cytokines and compete against environmental pathogens like *Staphylococcus aureus* via a secreted protease that cleaves *S. aureus* protein A and attenuates biofilm forming capability.^21^, ^22^ Once this barrier is perturbed, it can lead to infections from pathogens in the environment as well as skin commensals.^23^, ^24^ In PIs, these bacteria frequently form robust biofilms,^25^ in which microorganisms are encased in a matrix of self‐produced, extracellular polymeric substances (EPS) that typically consist of polysaccharides, nucleic acids, proteins and lipids.^6^, ^26^ Bacteria within biofilms are highly resistant and tolerant to the actions of the host immune system (e.g., avoiding detection and phagocytosis)^27^ as well as antibiotic treatment, making biofilm‐associated infections difficult to treat.^28^ In this context, it has been shown that biofilm cells are up to 1000 times more tolerant to antimicrobial agents than their planktonic counterparts. Several factors are associated with this increase in antimicrobial tolerance: low growth rate, high cell density, presence of persister cells, nutrient and oxygen gradients, EPS matrix, efflux pumps and horizontal gene transfer.^29^, ^30^ Biofilm formation is a cyclic process that includes three main events: the attachment of bacterial cells, biofilm growth and recruitment of surrounding cells, and the detachment of single bacteria or aggregates from the biofilm (Figure 2). In this updated biofilm model, bacteria can enter into the process at any given point, providing a more dynamic cycle that explains most biofilms, including those found in the clinic.^31^ Biofilms in PI can either be composed of a single species or are more frequently polymicrobial, encompassing a varying number of species ranging from a few microbes to several dozen, but typically contain between 2 and 20 different bacterial species.^32^ It has now been well established that biofilms are a major contributor to delayed wound healing and the development of chronic wounds.^33^, ^34^

## 3 PRESSURE INJURY MICROBIOME

Emerging evidence suggests that the composition and structure of the wound microbiome play an important role in chronic wounds.^33^ However, research on the PI microbiome has shown only some consistency in the bacterial profiles observed, specifically in relation to the abundance of each genus. For instance, Ammons et al. observed that these wound microbiomes consist predominantly of Firmicutes (40%), Proteobacteria (24%) and Actinobacteria (19%).^35^ In comparison, Dunyach‐Remy et al. showed different compositions of the three phyla where Firmicutes was far more dominant (80%) and Proteobacteria (6.5%) and Actinobacteria were less prominent (0.98%).^36^ In a recent study, Wettstein et al. analysed PIs from individuals with spinal cord injury to show that skin and PIs exhibited varying proportions of phyla, with Firmicutes and Actinobacteria being predominant in the skin, whereas Proteobacteria was the prevailing phylum in the PIs.^24^ Of note, recent studies have also indicated that the microbiome within PIs might vary with the depth and stage of the wound,^24^, ^36^ however, further studies are still necessary to completely understand the relationship between location within the wound, stage of the PI, and the associated microbial population. Nevertheless, within the Firmicutes phyla, most studies identified *Staphylococcus*, *Streptococcus*, *Enterococcus*, *Finegoldia* and *Anaerococcus* spp. as the commonly identified genera; Proteobacteria yielded *Proteus*, *Pseudomonas* and *Escherichia* spp., and *Corynebacterium* was the most common genus of Actinobacteria detected within these wounds.^24^, ^32^, ^35^, ^36^, ^37^, ^38^, ^39^, ^40^, ^41^, ^42^ Table 1 summarises common bacterial and fungal species identified in different PI studies. The involvement of fungi in chronic wounds has recently been reviewed by Ge and Wang^43^ and will therefore not be discussed in more detail here.

**TABLE 1:** Summary of most common bacteria and fungi in PI.

Aerobes/facultative anaerobes	Anaerobes
Gram‐positive bacteria	
*Actinomyces europaeus*	*Allobaculum* spp.
*Arcanobacterium haemolyticum*	*Anaerococcus lactolyticus*
*Brevibacterium antiquum*	*Anaerococcus vaginalis*
*Corynebacterium jeikeium*	*Blautia producta*
*Corynebacterium striatum*	*Clostridium hathewayi*
*Corynebacterium tuberculostearicum*	*Clostridium perfringens*
*Delftia acidovorans*	*Eggerthella lenta*
*Enterococcus faecalis*	*Eubacterium dolichum*
*Helcococcus kunzii*	*Finegoldia magna*
*Lactobacillus zaea*	*Gemella* spp.
*Mycobacterium vaccae*	*Peptococcus niger*
*Staphylococcus aureus*	*Peptoniphilus harei*
*Staphylococcus epidermidis*	*Peptoniphilus harei*
*Staphylococcus haemolyticus*	*Peptoniphilus indolicus*
*Staphylococcus lugdunensis*	*Peptoniphilus indolicus*
*Streptococcus agalactiae*	*Peptoniphilus ivorii*
*Streptococcus constellatus*	*Peptoniphilus lacrimalis*
*Streptococcus dysgalactiae*	*Ruminococcus bromii*
*Streptococcus mitis*	*Ruminococcus gnavus*
*Streptococcus parasanguinis*	*Varibaculum cambriense*
*Streptococcus thermophilus*	
Gram‐negative bacteria	
*Acinetobacter baumannii*	*Bacteroides fragilis*
*Alcaligenes faecalis*	*Bacteroides fragilis*
*Delftia acidovorans*	*Christensenella* spp.
*Enterobacter hormaechei*	*Dialister invisus*
*Escherichia coli*	*Dialister* spp.
*Ferrimonas* spp.	*Fusobacterium nucleatum*
*Flavobacterium succinicans*	*Parabacteroides distasonis*
*Hydrogenophaga* spp.	*Porphyromonas somerae*
*Klebsiella granulomatis*	*Porphyromonas uenonis*
*Pectobacterium* spp.	*Prevotella bivia*
*Pelomonas saccharophila*	*Prevotella buccalis*
*Proteus mirabilis*	*Prevotella* spp.
*Pseudomonas aeruginosa*	*Veillonella* spp.
*Riemerella* spp.	
*Serratia marcescens*	
*Serratia marcescens*	
*Shewanella* spp.	
*Stenotrophomonas maltophilia*	
Fungi	
*Aureobasidium pullulans*	
*Candida albicans*	
*Candida parapsilosis*	
*Cladosporium herbarum*	
*Curvularia lunata*	
*Malassezia restricta*	
*Malassezia sympodialis*	

An earlier study by Dowd et al. showed unique differences in PIs when surveying the microbiota, showing that 62% of the identified microbial community were anaerobes like *Peptoniphilus* spp. and *Peptococcus niger*, among other species.^39^ The colonisation of these anaerobic bacteria might suggest they play a significant role in the development of these wounds. The presence of anaerobic species like *Finegoldia* and *Anaerococcus* was associated with worse wound outcomes and, therefore, is a key microbe to consider when assessing treatment options. Furthermore, these anaerobes were found to be in close association with other microbes to enable a symbiotic relationship; facultative anaerobes utilise oxygen within the environment, leading to anoxic conditions and creating a localised niche where only strict anaerobes can persist. Examples of such associations are *Serratia* spp., a facultative anaerobe, in association with other obligate anaerobes such as *Finegoldia* and *Anaerococcus* spp.^36^ Another means by which these associations may impair wound healing is the interaction of strict anaerobes with immune cells, specifically inhibiting and degranulating polynuclear neutrophils (involved in ischaemia response and first‐line defence against infections).^36^, ^44^ In addition, the ischaemic environment within PIs can lead to a drop in oxygen, creating a local hypoxic environment.^45^ This environment can accelerate the growth of anaerobic microorganisms like *Finegoldia magna*, which can produce the subtilisin‐like serine proteinase *SufA* that can degrade collagen IV and collagen V and can afterwards break through the basement membrane of the skin, leading to deeper dermal tissue infections.^46^

Commensals such as *Corynebacterium* and *Staphylococcus epidermidis* have been shown to influence the healing within chronic wounds as they may develop synergetic interactions or have protective functions inhibiting colonisation of the wound with pathogens.^36^, ^47^, ^48^ These bacterial species are normally found on the dermis but may become pathogenic and cause infection if they are able to breach the skin barrier and if suitable conditions favour the proliferation of these bacteria.^49^ Additionally, commensals like *Corynebacterium* spp. can interact with other pathogens like *S. aureus* within the wound by regulating virulence factor expression in *S. aureus*.^50^ A recent report indicated the wound healing effects of *Alcaligenes faecalis*, a commensal observed in lymphoid tissues, by stabilising the expression of matrix metalloproteinase (MMP). MMP is detrimental due to its proteolytic nature and is highly abundant in delayed wound healing in diabetic foot ulcers.^51^ Other commensals that have been observed within PIs include *Enterococcus* and *Eubacterium dolichum*.^38^ As commensals occupy a niche in the microbiome of PIs, the species observed can vary with wound location as the skin microbiome displays spatial differences in the bacterial species observed (e.g., the glabella site is predominantly *Propionibacterium* spp. unlike the plantar heel which is occupied primarily by *Staphylococcus* spp.).^52^ In this context, sacral pressure injuries have recently been shown to contain more predominant gut‐related bacteria (e.g., *Escherichia coli* and *Enterococcus faecalis*) and fewer skin commensals due to faecal contamination.^41^, ^42^

Overall, the microbial diversity and how these microbiomes relate to PI disease progression remain poorly understood. Additionally, the mechanisms of how the presence or absence of pathogenic bacteria contributes to the onset, progression, and recurrence of PI remain elusive. In addition, since chronic wounds are naturally colonised by commensal bacteria, an important consideration for clinicians is to differentiate between colonisation and infection with the purpose of optimising the protective and wound‐healing functions of specific commensal bacteria.^36^ To further understand the biology of PI microbiomes and their contribution to chronic wound formation, in vitro and in vivo models mimicking the PI environment have been developed to study microbe–microbe and host–pathogen interactions and to evaluate potential novel treatment options.

## 4 IN VITRO BIOFILM MODELS

About 60% of chronic wound infections have biofilms, wherein *Pseudomonas* and *Staphylococcus* species are primarily observed.^32^, ^53^ To mimic the wound environment in the laboratory, external factors (e.g., nutrients and temperature) must be controlled to simulate natural conditions. Several in vitro biofilm models have been proposed to study chronic wound biofilms; however, despite being more than a decade old, the most promising model is the Lubbock Chronic Wound Biofilm (LCWB) model, which involves Bolton broth consisting of heparinised bovine plasma and laked blood simulating the nutrients in the wound environment.^54^ Sun et al.^54^ developed this LCWB model by using biofilm formation media (i.e., Bolton broth with 50% plasma and red blood cells) to replicate the wound environment and establish multispecies biofilms between three *ESKAPE* pathogens, *Pseudomonas aeruginosa*, *S. aureus* and *E. faecalis*. Additionally, a pipette tip was ejected into the inoculated media, which served as a surface to enable biofilm formation.^54^ In addition, Sun and colleagues used LCWB to establish a biofilm model growing anaerobic microorganisms such as *Clostridium perfringens*, *Peptoniphilus ivorii, Peptostreptococcus anaerobius*, *Anaerococcus lactolyticus* and *Finegoldia magna* together with the *ESKAPE* pathogens.^55^ Interestingly, it allowed for anaerobic organisms to grow under aerobic conditions within polymicrobial biofilms. Despite the findings of Sun et al.,^55^ the utilisation of the LCWB model to cultivate mixed biofilms of both anaerobic and aerobic bacteria has not been implemented very often. Regardless of this model being able to maintain a polymicrobial biofilm including aerobic and anaerobic bacteria, the method of transplantation of in vitro biofilms to a mouse does not accurately mimic how biofilms form and how bacteria adapt within the host. Another limitation with this model is the inaccurate concentrations of blood and plasma, which do not correlate with the values from human wounds.

In 2012, another biofilm model system was developed by Ngo et al.^56^ Their study surveyed the effects of topical negative pressure on wound biofilms. Using a bioreactor, agar, and in‐flow of nutrients like tryptic soy broth, these biofilm models can be established to examine microbial behaviour. This model demonstrated stable biofilms of *P. aeruginosa*, which were maintained for 2 weeks, determined by bacterial counts that stayed relatively the same throughout 14 days.^56^

The colony drip‐flow reactor (DFR) model was developed as a biofilm model to enable anaerobic species to proliferate and be examined. The DFR model involves a slide containing an absorbent pad with a 0.2 μm polycarbonate membrane on top where bacteria are inoculated and fresh nutrients flow through. For example, Woods et al.^57^ utilised the DFR model to grow polymicrobial biofilms that included a mixture of clinical isolates from PIs of *P. aeruginosa*, *S. aureus* and *C. perfringens*.^38^ The biofilms were supplied with BHI supplemented with adult bovine serum (5%) at a rate of 5 mL/h per channel for 3 days. They observed utilising light microscopy that each organism occupied specific niches within the model, where *P. aeruginosa* was found at the air–liquid interface and *S. aureus* and *C. perfringens* were found deeper in the anaerobic environment. This may reflect what occurs within PIs where both anaerobic and aerobic bacteria are often retrieved. The DFR is said to simulate some chronic wound characteristics such as wound exudate perfusing to the surface and maintaining contact with air.^58^

There remains a lack of studies examining the interactions of anaerobes and aerobes within chronic wounds, including PIs. We emphasise the importance of investigating the interaction of anaerobes with aerobes in polymicrobial biofilms of PIs to truly understand the impact on wound healing. Although in vitro studies do not perfectly recreate the natural environment where PIs occur (e.g., tissue organisation), these studies are valuable in studying PIs since they do not require the same ethical approval and costs required for in vivo studies.^59^

## 5 IN VIVO MODELS

To study the biology of PIs and PI infections, several in vivo models have been developed. While we describe some of these in vivo models in more detail below, we also provide additional examples for in vivo models studying PIs, PI infections, as well as wound healing studies on PIs in Table 2 and Table 3.

**TABLE 2:** Examples for in vivo PI models without infection.

Reference	Model description
Peirce et al.^60^	Initial rat model, dorsum implanted with ferro magnetic steel plate in fascia and pressure application 50 mm Hg. I/R cycles varied from 2 h ischaemia, followed by 0.5 h reperfusion, 2 h ischaemia, followed by 1 h reperfusion. Maximum 5 compression (ischaemia) cycles followed by 11.5 h reperfusion conducted for
Stadler et al.^62^	Initial mouse model utilising two magnets sandwiching skin, pressure application at 50 mm Hg. I/R cycles of 12 h ischaemia
Wasserman et al.^64^	Nude mice model with implanted steel disc under great gluteus muscle. I/R cycles included 2 h ischaemia, 1 h reperfusion
Maldonado et al.^111^	Diabetic/severe combined immunodeficiency mice were engrafted with human skin. Pressure applied at 150 mm Hg. I/R cycle, 8 h ischaemia, 16 h reperfusion for 3 cycles
Takeuchi et al.^112^	Mouse model implanted magnet under skin sandwiched between external magnet for 7 consecutive days, produced large amounts of exudate and induced inflammatory cell infiltration of PI
Kumar et al.^113^	Mice underwent laminectomy, complete spinal cord transection (T9‐T10 vertebrae), skin fold lifted and sandwiched between two magnets for 12 h, PI developed over subsequent days
Sami et al.^114^	Streptozocin induced diabetic mice with implanted magnet deep into panniculus carnosus muscle, sandwiched with external magnet for 5–7 days. Deep ulcer in subcutaneous tissue (stage III PI)
Kwek et al.^115^	A pair of magnets was used to sandwich mouse skin. I/R cycle 1.5 h ischaemia hours followed by 24 h reperfusion created

**TABLE 3:** Examples of in vivo models that investigated wound healing of PI in non‐infection and infection models.

Study	Model description
Saito et al.^116^	Loss of monocyte chemoattractant protein 1 (MCP‐1) attenuated cutaneous I/R injury in PI mouse model
Assis De Brito et al.^117^	Propanol impaired closure of PI in mice
Uchiyama et al.^118^	Secreted glycoprotein and integrin‐ligand MFG‐E8 promoted cutaneous wound healing of I/R injury by enhancement of angiogenesis
Fang et al.^119^	Inhalation of hydrogen gas protected against cutaneous I/R injury in mice model of PI
Alexadrushkina et al.^120^	Multipotent mesenchymal stomal cells (MSC) delivered by injection induced wound healing in PI in mice. MSC prevented fibrosis by triggering effects of granulation tissue and vascularisation in mice
Thome Lima et al.^121^	Photobiomodulation accelerated wound healing in mice infected with *Pantoea agglomerans*
Yamazaki et al.^122^	Apelin/APJ signalling suppressed PI formation
Perez‐amodio et al.^123^	Polymeric composite dressings containing calcium‐releasing nanoparticles accelerated wound healing in type II diabetic mice
Toita et al.^124^	Apoptotic mimic‐induced M2‐like macrophages polarisation on PI in young‐middle aged mice had protective and healing effects Phosphatidylserine containing liposome (PSL) induced M2 macrophage polarisation. PSL signalling inhibited PI formation and promoted tissue
Wano et al.^125^	Whole body vibration attenuated wound inflammation and enhanced collagen deposition in stage II PI in mice.
Huang et al.^126^	Chitooligosaccharide‐europium functional micron complex facilitated visual inflammation monitoring via fluorescence property pH sensitivity. Exhibited anti‐inflammatory activity by synergising with Eu to promote vascularisation and tissue regeneration
Menegasso et al.^127^	Bacterial cellulose hydrogel incorporated into montmorillonite healed PI in mice exhibited by reduced redness, spontaneous hyperalgesia, lower amounts of inflammatory cells and complete epidermis re‐epithelialisation and tissue regeneration
Ohta et al.^63^	Silver‐loaded carboxymethyl cellulose, controlled counter ions accelerated PI wound healing in *P. aeruginosa* infected mice

In 2000, one important in vivo PI model was developed by Peirce et al. using ischaemia–reperfusion skin injury in rats, characterised by wound thickness, tissue necrosis, leukocyte infiltration, transcutaneous oxygen tension and wound blood flow.^60^ This model used an implanted ferromagnetic steel plate (9 cm^2^) nested in the fascia, where rats were subjected to ischaemia and reperfusion (I/R) cycles using a magnet (4.0 × 2.25 × 1 cm, 122 g, 1250 G) that applied pressure at 50 mm Hg (clinically relevant based off the pressure interface between the greater trochanters of volunteers and hospital replacement mattresses). The I/R cycles were performed when rats were anaesthetised with varying ischaemia and reperfusion times, such as 2 h of ischaemia followed by 1 h of reperfusion (based off the clinical recommendations for patients at risk of developing PIs should be turned/repositioned at least every 2 h). This in vivo PI model is highly adaptable and can be catered to varying PIs by characteristics and stages, altering the length of the I/R cycles. In this context, the Peirce et al.^60^ rat model has recently been used for assessment of the effects of atmospheric pressure and cold plasma on tissue healing as well as effects of antimicrobials on wounds infected with *P. aeruginosa*.^61^ Rats with stage III PIs had a 10^8^ CFU of a multi‐drug resistant clinical isolate of *P. aeruginosa* introduced into the wound. However, this model had several limitations, including that not all PIs were successfully infected. Therefore, the authors injected bacteria directly into the PI, and tissue samples were taken after 24, 48, 96 and 144 h to ensure successful infections. Rats were treated with atmospheric pressure cold plasma (APCP), silver sulfadiazine (AgS) or saline once a day for 14 days. Intriguingly, APCP reduced the surface area and depth of infected PIs and exhibited epithelialization superior to that of AgS and saline. Furthermore, on day 15, bacterial loads dropped from 12 log_10_ CFU/g to 5.64 log_10_ CFU/g of tissue. Infection via dropping bacteria onto PIs was inconsistent; the best method of infection was via direct injection of the bacteria into the PI site. It is, however, questionable how well a direct bacterial delivery reflects the natural development of an infected ulcer.

Stadler et al.^62^ further adapted the Peirce model in mice using smaller magnets and without the implantation of a magnetic steel plate into the fascia. Instead, mouse skin was pinched (5 mm skin bridge) between two magnets (12 mm diameter, 5 mm thick) and pressure was applied at 50 mm Hg. The I/R cycles consisted of 12 h of compression (ischaemia) followed by 12 h of release (reperfusion) across 3 days (3 cycles). This model was recently used for 6 days before debridement and the inoculation of *P. aeruginosa* (10^6^ CFU) on top of the PI^63^ to examine the effects of silver‐loaded carboxymethyl cellulose on wound healing.

Wasserman et al.^64^ modified the Peirce et al.^60^ model and developed a model that induced stage IV PIs in nude mice (Balb/c). This involved a steel disk implanted under the great gluteus muscle before pressure was applied across 1, 4, 6, 8 and 10 cycles of 2 h (ischaemia) with 1 h recovery (reperfusion). The number of cycles determined the grade of the ulcer, where stage I PIs formed after 4 cycles, stage II formed after 6 cycles to stage III after 8 cycles and stage IV after 8–10 cycles. The advantage of this model is that it can be easily adapted to simulate different PI stages (from I to IV) while also taking advantage of the immunodeficiency and absence of hair. Similar to the Peirce et al.^60^ model, the main disadvantage is the invasive nature of the implantation of the steel magnet. This Wasserman et al.^64^ model was used to induce stage IV PI (2 h ischaemia, 1 h reperfusion for 10 cycles) in TALLYHO/JngJ mice.^65^ This model introduced methicillin‐resistant *S. aureus* 1 day after PI formation using a high density of 10^9^ CFU, followed by auranofin, mupirocin, or clindamycin treatment 2 days post infection. Intriguingly, auranofin completely eradicated *S. aureus* upon topical treatment after 4 days, which was superior to mupirocin and clindamycin.

Common among PI infection models is the use of only one species of bacteria, none of which investigate anaerobic species. The lack of studies on polymicrobial‐infected PI and treatment is a problem, since most PI infections harbour a diverse range of microorganisms. There is an urgent need for PI infection models that use multiple different species of bacteria, including anaerobic species. Chronic wound infection models involving anaerobic bacteria are scarce, which leaves a huge gap in our knowledge of what role they play in PI infections and how they may interact with the host. The presented studies suggest that infecting mice or rats before or during the middle of ischaemia/reperfusion cycles is potentially more accurate to simulate how infection may occur and progress within PI.

## 6 TREATING PRESSURE INJURIES

There are a number of treatment options that modify the wound environment when infection develops. Wound dressings, antimicrobial chemotherapy, and negative pressure therapy are commonly used non‐invasive options.^66^ Wound dressings are selected for their ability to inhibit bacterial contamination, provide pain relief, prevent water vapour loss (important in moisture balance), and absorb exudates.^66^ Different types of dressings are available, although protease‐modulating dressings, foam dressings and collagenase ointments are prominent choices found to help heal wounds faster than gauze and other ointments.^67^ Topical agents can also be employed before applying the wound dressing to promote recovery (i.e., granulation and epithelization).^68^ Examples of such agents used in PI treatment include collagenase‐containing ointment and hydrogels.^69^ Wound dressings and topical agents are believed to produce a moist environment within the wound, allowing for autolytic debridement of the necrotic tissue and therefore leading to the development of healthy new skin.^70^ If the infection is deep and/or sepsis has developed, systemic antibiotics are required, often in tandem with surgical debridement.^67^, ^71^ Antibiotics should be administered if there are clinical signs and symptoms of deep wound infection. Commonly prescribed antibiotics include macrolides, penicillins, quinolones and lincosamide antibiotics.^72^ Due to the presence of biofilms and the increasing amount of multi‐drug resistant bacteria globally, alternatives to systemic and topical antimicrobials are sought after; for instance, negative pressure therapy and debridement. Negative pressure therapy promotes healing by applying negative pressure to extract fluids and exudates and assists in stimulating granulation to close the wound.^72^ The presence of necrotic tissue in the wound is a factor thought to delay wound healing; therefore, debridement is used to remove necrotic tissue, aiming to prevent further complications like sepsis.^71^ Surgical debridement is the most effective way of removing the dead tissue layers; however, other types of debridement can be implemented. However, if the infection of these wounds has developed and the aforementioned strategies are not sufficient to stop the progression of the wound, novel treatments are required to reduce bacterial burden.

## 7 ALTERNATIVE TREATMENT APPROACHES

The presence of biofilms within infected PI and chronic wounds in general, and the rise of antibiotic resistance, demands the development of new treatment and delivery strategies. In this context, a recent study by Vasconcelos et al.^73^ collected wound swabs from the PIs of patients at the university hospital in Rio de Janeiro, Brazil and concluded that 91.7% of bacterial strains obtained from PI patients were resistant to *at least* one of the antibiotics recommended by Clinical and Laboratory Standards Institute guidelines [2022]. In similar studies, ~64% of the wounds screened in a PI population were found to contain one or more multi‐drug resistant organisms.^74^, ^75^

Alternative approaches include the administration of antimicrobial peptides, the application of hydrogel wound dressings, or a combination of antimicrobials encapsulated in a hydrogel to improve wound healing and reduce the bacterial burden. Antimicrobial peptides (AMPs) are host defence peptides that are a promising novel strategy for combatting antimicrobial resistance due to their broad‐spectrum activity against both Gram‐negative and Gram‐positive bacteria.^76^ AMP, human cathelicidin LL‐37 produced by the immune system, has multiple roles including acting as an alarmin (cell and tissue damage signal) to induce an immune response, modulating the inflammatory response, promoting wound healing, and possessing direct antimicrobial and antibiofilm activity against pathogenic intruders.^77^, ^78^ Due to LL‐37 having low stability within the wound microenvironment, methods have been undertaken to improve this including loaded nanocarrier^79^, ^80^ and synthetic modifications.^81^, ^82^, ^83^, ^84^ Hydrogels are 3D networks of hydrophilic polymers that create a colloidal gel in a water‐rich environment.^85^ Yang et al.^86^ mixed chitosan hydrogel (2.5% w/v) with LL‐37 to create chitosan hydrogel encapsulated with LL‐37 (LL‐37/CS) and assessed the antimicrobial activity against *S. aureus* and the activity on a wound healing of a deep tissue injury in a mouse model (similar to Stadler et al.^62^ PI model). At 5 μg/mL, LL‐37/CS inhibited *S. aureus* growth. Furthermore, mice treated with LL‐37/CS induced the expression of macromolecules involved in angiogenesis in the wound tissue, and PIs were significantly smaller in size. This study highlights the potential hydrogels in combination with AMPs have for the treatment of PI infections. For a more comprehensive review on host defence peptides and their impact on chronic wounds, see Haney et al.^87^, ^88^

Hydrogels are not only used and investigated in drug delivery, but they are also being investigated for the enhancement of wound dressings.^89^, ^90^ In this context, chitosan‐based wound dressings and hydrogels have emerged as another option for chronic wound treatments.^89^, ^90^ Chitosan is a natural cationic polysaccharide polymer that exhibits antimicrobial, biodegradable, and non‐toxic properties and also promotes wound healing.^91^ In a recent study, chitosan was crosslinked with polyethylene glycol diacid hydrogels and the antibacterial, anti‐inflammatory, and self‐healing activities of these hydrogels were shown.^92^ Furthermore, in a 30‐day clinical pilot study, 20 adult volunteers with PI were given chitosan gels to show that 90% of patients were treated effectively with a reduction of the area of the lesion and wound healing progress.^93^

In addition to AMPs and hydrogels, several advanced alternative treatment options like phage therapy^94^, ^95^ for PI have been investigated and tested via controlled clinical trials to confirm their safety and efficacy.^96^ Although this alternative shows promise, its limitations prevent it from being a viable option. Such limitations include the narrow specificity of phages only targeting specific bacterial genera, whereas others remain relatively untouched; the lack of clinical policies and applications, whereby no standard framework for phage isolation and preparation affects the efficacy of these treatments. Other limitations include resistance to such bacteriophages, insufficient data on the mode of administration and pharmacokinetics, and unwanted immune reactions.^97^ Among them, the supply of oxygen to the wound to accelerate key wound‐repairing processes through transdermal wound oxygen therapy (TWOT) and HBOT has been extensively evaluated.^96^ Since hypoxia is known to delay the healing process, studies have shown that the administration of oxygen to wound sites has been successful in wound management.^98^ Oxygen is critical for wound healing since it promotes cell proliferation, synthesis of collagen, angiogenesis, and suppresses bacterial growth by producing reactive oxygen species.^99^, ^100^ Azimian et al.^96^ analysed the effect of TWOT on the healing of PI by evaluating 100 patients with PI in sacral and ischial areas and showed that TWOT decreased the wound area significantly by the eighth and twelfth day (mean of 13.36 ± 7.07 cm) compared with the patients that did not receive the treatment (mean of 31.81 ± 3.94 cm). Furthermore, a higher number of patients with completed healing were observed after the treatment with TWOT (80%) compared with the control group (35%).^96^

HBOT consists of the inhalation of pure oxygen at a constant pressure in a compression chamber to increase the supply of oxygen to the tissues. HBOT has been used to treat several health conditions, for example, carbon monoxide poisoning, air embolism, thermal burns, necrotising soft tissue infections and chronic refractory osteomyelitis.^101^ HBOT has also been used to treat diabetic foot ulcers.^102^ The main effect of HBOT is attributed to the increased level of reactive oxygen and nitrogen species, which increase the synthesis of growth factors, stem cell mobilisation, fibroblast proliferation and collagen production favouring the healing of wounds.^103^ Recently, HBOT showed promising results in an in vivo study performed by Laulund et al. when chronic wounds in mice infected with *P. aeruginosa* were treated with HBOT in addition to ciprofloxacin.^104^

Overall, although the results of using oxygen to promote the complete healing of chronic wounds are promising, evidence remains not sufficient and in‐depth studies on the efficacy and safety of these treatment strategies must be conducted. Furthermore, clinical trials with a larger sample size, standard and well‐described methodological approaches should be conducted to evaluate not only the short‐term but also the long‐term effects of these therapies on wound healing.

In summary, new treatment methods typically either focus on wound healing or reducing the bacterial burden of the infection, but usually not at the same time. However, this is important to consider because the bacterial burden within PI infection interferes with wound healing processes. We believe that there is a lot of potential for combinations of wound healing agents and antimicrobial compounds. Such bioactive compounds can be incorporated in cellulose‐based wound dressings, which were recently reviewed by Firmanda et al.^105^ Due to the important role that polymicrobial biofilms play in PI infections, we also emphasise the need to evaluate novel treatment combinations using polymicrobial biofilms in different model systems.

## 8 FINAL REMARKS

With the prevalence of PI at a high and the increasing expenses to treat these wounds, there is an urgent need to better understand the contribution of biofilms to chronic wound formation in PI to improve treatment methods. In recent years, there has been more attention dedicated to developing sophisticated PI model systems for both in vivo and in vitro studies. These models will be vital in elucidating mechanisms involved in the aetiology of PI, the roles of biofilms in host–pathogen interaction in PI infections, and to recognise how the PI microbiome contributes to chronic wound formation and delayed wound healing. Many studies have utilised various patients with differing PIs to identify the microbial composition of these wounds, but no distinct publication has reported the microbiota at each stage. One journal published a review of various studies that examined the microbiome of PIs; however, the majority of studies were conducted using specific culture methods and/or individuals that met their inclusion criteria (Stage 2 PI or higher).^106^ New technologies such as metagenomic shotgun sequencing, Oxford nanopore sequencing, and advanced fluorescence wound imaging, which have recently been used to analyse diabetic foot ulcers,^107^, ^108^, ^109^ will be valuable tools in the study of PI infections in the future. Nanopore sequencing and other metagenomic approaches have been utilised within DFUs to characterise anaerobic bacteria and underrepresented microbes (including fungi) to depict a more comprehensive bacterial profile of these wounds.^108^, ^110^ With antimicrobial resistance on the rise, several promising new treatment approaches are currently under investigation; however, these will still need more time to find their way into the clinics. Despite the efforts of more than 20 years of biofilm research in PI, more studies are required to better understand the complex interactions between biofilms, the PI microbiome, and the host immune system to improve patient outcomes.

## AUTHOR CONTRIBUTIONS

All authors contributed to the article and approved the submitted version.

## FUNDING INFORMATION

This work was supported by a Bruyère Academic Medical Organisation (BAMO) Innovation Grant, a Carleton University Research Development Grant, and an International Research Seed Grant.

## CONFLICT OF INTEREST STATEMENT

The authors report that there are no competing interests to declare.
